# Development of a Predictive Model of Cardiovascular Risk in a Male Population from the Peruvian Amazon

**DOI:** 10.3390/jcm12093199

**Published:** 2023-04-29

**Authors:** Jose M. Alcaide-Leyva, Manuel Romero-Saldaña, María García-Rodríguez, Rafael Molina-Luque, Rocío Jiménez-Mérida, Guillermo Molina-Recio

**Affiliations:** 1 Departamento de Enfermería, Farmacología y Fisioterapia, Facultad de Medicina y Enfermería, Universidad de Córdoba, 14014 Córdoba, Spain; 2 Grupo Asociado de Investigación GA16 Estilos de Vida, Tecnología y Salud, Instituto Maimónides de Investigación Biomédica de Córdoba (IMIBIC), 14014 Córdoba, Spain; 3 Departamento de Enfermería y Nutrición, Facultad de Ciencias Biomédicas y de la Salud, Universidad Europea de Madrid, Calle Tajo S/N. 28670, Villaviciosa de Odón, 28670 Madrid, Spain

**Keywords:** predictive model, cardiovascular risk, non-communicable disease, obesity and Peruvian Amazon

## Abstract

Background: The coexistence of malnutrition due to over- and under-nutrition in the Peruvian Amazon increases chronic diseases and cardiovascular risk. Methods: A cross-sectional study of a male population where anthropometric, clinical, and demographic variables were obtained to create a binary logistic regression predictive model of cardiovascular risk. Results: We compared two methods with good predictive results, finally choosing Model 4 (r^2^ = 0.57, sensitivity 73.68%, specificity 95.35%, Youden index 0.69, and validity index 94.21), with non-invasive variables such as blood pressure (*p* < 0.001), hip circumference (*p* < 0.001), and FINDRISC test result (*p* < 0.05); Conclusions: We developed a cheap, fast, and non-invasive tool to determine cardiovascular risk in the population of this endemic area.

## 1. Introduction

The coexistence of under- and over-nutrition is known as the double burden of malnutrition [1]. Since it is related to many pathologies derived from these two nutritional statuses, the double burden of malnutrition is becoming an emerging crisis for all middle- and low-income countries [2]. Overweight and obesity have increased substantially in these countries, reaching a prevalence of 21.1% and rising even more in Peru, to 47.9% [3]. This problem is due to the changes in food patterns that occurred throughout South America, where traditional food models based on pre-Columbian cultures evolved into Western ones. These changes increase high blood pressure (HBP) and Type 2 diabetes mellitus (DMT2) and dyslipedemia rates [4]. This situation is made worse because of the constant migration from rural areas to urban peripheries, where the prevalence of other risk factors such as illiteracy, violence, stress, or malnutrition show higher figures [5].

The prevalence of HBP in Latin America is 45.5%, and in Peru it reaches 14.5%, with a rate of 26.6% in the jungle, according to the TORNASOL II study [6]. Regarding DMT2, in the urban population of Latin America, the prevalence is between 4–8%. However, the data are relatively scarce and the percentage of patients without diagnosis is around 30–50%. In addition, it is estimated that the prevalence could be much higher in rural areas. In the case of Peru, this percentage of people with diabetes was 25.6% [4]. In terms of dyslipedemia, the data showed 17.4% hypercholesterolemia and 14.9% hypertriglyceridemia [7]. This research was conducted in Iquitos and specifically in Pueblo Libre, a slum located in the district of Belen that is characterized by periodic flooding between February and June. The geographical location of Iquitos confers a transitional character between the communities near the rivers of the jungle and the city itself [8].

Few studies have been conducted to evaluate the risk of DMT2, HBP, and dyslipidemia in this area, probably due to the difficulty in performing laboratory analysis and clinical tests in disadvantaged zones. These problems are linked to the unavailability of material and human resources and poor conditions [9]. For these reasons, we considered it necessary to develop a non-invasive, inexpensive, and easy-to-use diagnostic or screening method for these chronic conditions. This method could provide two advantages: (i) it does not require laboratory tests, and (ii) it makes it possible to identify which individuals at risk (with a positive result) could benefit from laboratory testing.

In 2003, Lindström and Tuomilehto proposed the FINDRISC questionnaire to predict the risk of developing DMT2 within ten years. Although it was designed for the residents of Finland, several studies have used it in different populations around the world, which shows that it is a flexible instrument for screening and preventing individuals at high risk of diabetes [10].

In 2016, our research group conducted a study among women of this population due to their high vulnerability [8]. Three years later, we decided to come back and study the other half of the citizens to develop a non-invasive method to predict cardiovascular risk (CR) and compare results.

## 2. Materials and Methods

A cross-sectional study was conducted in the slum of Pueblo Libre (Iquitos) from February to May 2019. The total number of residents was 6042. From these, we selected those over 16 years old, obtaining a total of 3017 subjects and, among them, 1424 men. Using Epidat 4.2, we determined that the sample size for absolute precision of 3%, a confidence interval of 95%, and expected prevalence of 3% and 11.3% for DM and HBP, respectively, was 329 participants. Therefore, a sample of 363 participants structured by sector within the population and age was selected.

1. Independent variables
  1.1 Anthropometric variables: height in centimeters, weight in kilograms, BMI (kg/m^2^), waist and hip circumference in centimeters, ABSI and BAI indices, and body fat percentage using Deurenberg’s equation [11].
  1.2 Analytical variables: capillary glycemia in mg/dL and systolic (SBP) and diastolic (DBP) blood pressure in mmHg.
  1.3 The eight items included in the FINDRISC questionnaire to predict DM^9^:
    - Age;
    - Previous diagnosis of T2DM in a family member;
    - Waist circumference (WC);
    - Physical activity of at least 30 min daily;
    - Frequency of vegetable intake;
    - Use of antihypertensives;
    - Previous high blood glucose level;
    - BMI.
   The final test scores ranged from 0 to 24 points, with the following interpretation:
    - <7 points: low risk level (1%);
    - From 7 to 11: light risk level (4%);
    - From 12 to 14: moderate risk level (17%);
    - From 15 to 20: high risk level (33%);
    - More than 20: maximum level of very high risk (50%).
  1.4 Sociodemographic variables: age, schooling level (no schooling, elementary, or higher education), employment situation (employed or unemployed), marital status (single, married, widowed, or divorced).
2. Outcome variables
  2.1 High blood pressure (HBP): following the VII JNC [12], HBP was considered when systolic blood pressure (SBP) reached 140 mmHg or higher and/or diastolic blood pressure (DBP) equaled or exceeded 90 mmHg.
  2.2 Diabetes mellitus: the authors applied criteria established by the American Diabetes Association for DM diagnosis [13]:
    2.2.1 Fasting plasma glucose values (without having ingested calories in the last eight hours) of 126 mg/dL or higher.
    2.2.2 Plasma glucose values equal to or greater than 200 mg/dL after two hours of an oral glucose tolerance test using 75 g of anhydrous glucose dissolved in water.
    2.2.3 Patients with classic symptoms of hyperglycemia or hyperglycemic crisis or a random plasma glucose ≥200 mg/dL.
  2.3 Cardiovascular risk: co-presence of a minimum of two of the following diseases: HBP, DM, or obesity.

The researchers followed the international standards for anthropometric assessment (ISAK) [14] to collect the anthropometric data. All measurements were performed by specifically trained staff, who took each measure three times to reduce the variation coefficients. Finally, the mean of these three measures was registered.

Weight was assessed to the nearest 0.1 kg using a Tanita BC545NSV20 electronic scale (Tanita, Tokyo, Japan). Then, a digital measuring stadiometer with an accuracy of 0.1 cm, model AC 1200D (Davi & Cia, Barcelona, Spain), was used to measure height.

A Lufki W606PM anti-elastic metallic tape (Lufki, Missouri City, TX, USA) with an accuracy of 0.1 cm was used to measure waist and hip circumference. For waist circumference, researchers took the midpoint from the inferior limit of the lower rib to the iliac crest. Regarding hip circumference, they placed the band around the hips to the level of the major trochanter. Both circumferences were measured at the end of a regular exhalation. Participants were upright, with both feet placed together and arms suspended next to the torso.

Arterial blood pressure was monitored using an OMRON M4 tensiometer (OMRON, Kyoto, Japan) [15] following a rest period of 10 min. Three readings were taken, waiting for a one-minute interval between them [16].

Lastly, capillary blood glucose was assessed with an Accucheck Vivo Active 3 (Roche, Basilea, Switzerland) digital glucometer, according to manufacturer guidelines [17].

### 2.1. Ethical Considerations

This study strictly followed the guidelines of the Declaration of Helsinki on ethical principles in medical research. All participants were personally, verbally and in written form, aware of the aims of the research study. Researchers also informed them of the hazards and advantages of their involvement in this project. All informed consents were signed and preserved.

### 2.2. Statistical Analysis

SPSS 22 software (IBM Corp., New York, NY, USA) was used for statistical analysis. The quantitative variables were expressed as mean and standard deviation, while the qualitative variables were shown as percentages. The parametric Student’s *t*-test or the nonparametric Mann–Whitney U test were applied to compare two means, depending on the normality of the data. For comparing three or more means, the variance test (ANOVA) analysis was used as a parametric test with the Bonferroni method for post-hoc contrasts. The Kruskal–Wallis test was used as a nonparametric test. The Kolmogorov–Smirnov test (*n* > 50) with the Lilliefors test correction and a graphical representation as a histogram or Q-Q and P-P plots were calculated to determine the goodness of fit to a normal distribution of the quantitative variables. For sample sizes of less than 50 individuals, the Shapiro–Wilk test was used to contrast the normality of the data. When necessary, a chi^2^ test and Fisher’s exact test were used to compare the percentages of qualitative variables.

Binary logistic regression was also computed, with the determination of the crude odds ratio (OR) values for each independent variable and adjusted ORs for the final variables of the model. The Wald test was applied as a statistical contrast test. The Hosmer–Lemeshow test was carried out to establish the model’s goodness of fit. Finally, the Cox–Snell and Nagelkerke deviance and determination coefficients were used to determine the model’s predictive validity.

We also performed the ROC (receiver operating characteristic) curves and each independent variable’s area under the curve (AUC) to determine the model’s predictive power. Finally, the sensitivity, specificity, positive and negative predictive values, and Youden index of the two final models were determined.

Researchers determined the significance level for an alpha error of less than 5% for all tests, and confidence intervals were calculated for a 95% confidence level.

## 3. Results

### 3.1. Population and Sample

The sample consisted of 363 men ranging from 18 to 84 years of age. In addition, 75.5% were married, and 76.6% had not studied or just had primary studies.

Regarding their nutritional status, the mean BMI was 26.5 (4.5) kg/m^2^; 42.3% showed healthy weight, 37.6% were overweight, and 20.3% were obese.

Table 1 shows a summary of the rest of the characteristics of the sample.

### 3.2. Bivariate Analysis and Logistic Regression of HBP, DMT2, and CR

Based on the demographic and anthropometric variables, the items included in FINDRISC, and the presence of different chronic diseases studied, associations were analyzed through bivariate analysis and logistic regression (Table 2, Table 3 and Table 4).

DMT2 was present in 1.6% (95% CI 0.2–3.1) of the subjects. No significant differences were found for demographic or anthropometric variables except for BAI (*p* < 0.05). Table 3 shows the results regarding HBP. The prevalence of this disease was 22.5% (95%CI 18.9–26.95%) (*p* < 0.001).

Only age obtained significant differences (*p* < 0.001) as a demographic variable. In the case of anthropometry, all variables showed significant differences. Among the FINDRISC questionnaire variables, differences were found; in FINDRISC, high/low (*p* < 0.001), >30 min daily physical activity (Q4) (*p* < 0.005), history of antihypertensive medication (Q6) (*p* < 0.001), and WC risk (Q3) (*p* < 0.001).

The results of the CR variable are shown in Table 4, in which the relationship to employment status stands out (*p* < 0.05), being more prevalent in the unemployed (10.3%). Differences were also found with nutritional status (*p* < 0.001), where the prevalence of CR in obese participants (39.2%) stands out. All the anthropometric variables obtained significant differences except for ABSI. The number of significant variables remained constant in the logistic regression.

### 3.3. Comparison of Adjusted Models and Diagnostic Accuracy for CR

Finally, four adjusted logistic regression models were calculated to predict CR, set up with the significant variables of the crude model (Table 5).

Out of the four models, models 1 and 4 were compared (Table 6), as they obtained a higher goodness of fit: r^2^ = 0.62 and r^2^ = 0.57, respectively. Model 1 included HBP as a qualitative variable (OR = 56.8 CI95% (15.15–214.21) (*p* < 0.001)), in addition to WC as a quantitative variable (OR = 1.26 CI95% (1.15–1.38) (*p* < 0.001)), and was excluded because of the wide confidence interval and the very high odds ratio.

Moreover, Model 4 included high/low FINDRISC as a qualitative variable and obtained an OR of 7.86 CI95% (1.42–43.5), as well as two quantitative variables: SBP (OR = 1.08 CI95% (1.05–1.12) (*p* < 0.001)) and HC (OR = 1.24 CI95% (1.14–1.35) (*p* < 0.001)).

**Figure 1:**
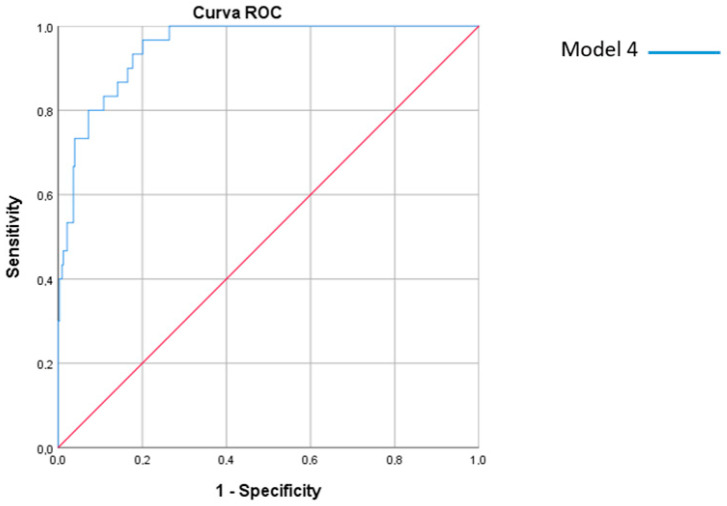
ROC curve for Model 4.

ROC curves were performed to determine the discriminant ability of Model 4 (Figure 1). From these, cutoff values were determined to calculate diagnostic accuracy indicators. Thus, our model achieved a sensitivity of 73.68%, a specificity of 95.35%, and a Youden index of 0.69. The validity index was 94.21%.

## 4. Discussion

During our second stay in this area, this screening was conducted to determine the prevalence of non-communicable diseases (NCDs) such as HBP and DM, nutritional status, and CR. This time, we decided to complete the study with the 363 men for whom we did not obtain data during our first stay. As a result, the prevalence found for overweight and obesity were 37.5% and 20.3%, respectively, being lower than those of 39.1% and 21.3% previously obtained in the last report published by the Peruvian Health Institute and its Food Surveillance System by Life Stages (VIANEV) [18].

Regarding chronic diseases, the prevalence found was 1.6% for DMT2 and 22.5% for HT, again lower than the national average for men of 12.5% and 33.6%, respectively. CR, our main outcome variable, which we define as the joint presence of two or more of these NCDs (DMT2, HBP, obesity), reached a prevalence of 8.24%, well below the 43% found for the South American and Caribbean population in the meta-analysis by Huaquía-Díaz et al. [19].

The difference in the prevalence of DMT2 is remarkable compared to the national average. In this sense, it was theorized whether the highly endemic population, with a percentage of indigenous individuals of 21.3% in the case of men in the province of Maynas, where Pueblo Libre is located, would have led us to obtain percentages different from the national or regional averages, in which all phenotypes and ethnicities are considered collectively [20].

Initially, a better health situation was found compared to the rest of the country. However, abdominal obesity determined through anthropometric measures such as WHtR and WHR were above average, with 0.56 and 0.96, respectively. This determined the existence of cardiovascular risk in the local citizens, as also reported by Paz-Krumdiek et al. for the population of the other areas of the Peruvian Amazon [21].

In addition, based on the previous experience of other authors with Mediterranean populations from Spain [22], we related the items that compose the FINDRISC test and its cutoff points with factors that predispose the presence or increase in CR. It was highly significant when the test scores were grouped dichotomously between high and low risk to be related to HT, T2DM, obesity, and CR.

### 4.1. Importance of a Predictive Model for the Amazonian Population

The prevalence of the different NCDs in Pueblo Libre is much lower than the data collected in national reports This fact indiates the need to modify the cutoff points of the scales or variables used in diagnosing CDNs, requiring an adaptation to the phenotypic characteristics of the population. Other authors have already evidenced this in other contexts [23]. In addition to the lack of adaptation of existing models, specifically for the characteristics of this community, there was a complete lack of development of predictive instruments for our population and any other group with similar characteristics. Rodrigo M. Carrillo-Larco et al. [24] emphasized the same deficit in their research, which went beyond the countries bordering northern Peru to the rest of Latin America and the Caribbean.

Considering this deficiency, we developed several predictive models for CR based on logistic regressions that included the study’s independent variables. Among these, Model 4 stood out, incorporating quantitative variables such as SBP, HC, and the dichotomous qualitative variable low/high FINDRISC. This model showed an adjusted coefficient of determination R2 = 0.58, a Youden index of 0.69, 73.68% sensitivity, and 95.35% specificity.

In other words, we believe that the proposed model improved de facto the predictive capacity of the CR for the Amazonian population. Furthermore, since it had only three explanatory variables obtained by interview and with non-invasive and manual techniques, the simplicity of its applicability stands out. Moreover, any health professional could implement the proposed model in any health context (hospital, community, educational, military, or social). However, this non-invasive approach could sidestep the reality of the leading health policies in most low- and middle-income countries since there is a gap between the development of laws that include tools for controlling NCDs and their actual applicability in the target population [25,26].

### 4.2. Comparison with Other Models

Previous models developed to predict CR included behavioral, and therefore subjective, variables and laboratory-analyzed variables that required a blood sample and a more significant number of resources and expenditures. These were easily applicable in high-income countries where access to private or public health care resources was readily available. Such is the case of the model carried out by Li Yang et al. with an AUC = 0.781 primarily based on clinical variables such as HDL cholesterol, LDL, triglycerides, basal blood glucose, etc. [27]. On the other hand, there were models obtained through artificial intelligence, such as machine learning, using information from databases that contain complete medical histories (previous diseases, treatments, surgical tests, diagnostic tests, etc.) from patients with no prior CR that obtained an AUC = 0.781 [28]. Even the Framingham test, widely used by the scientific community and with an AUC = 0.721 [29], included data on dyslipidemia that would be difficult to obtain in a setting such as the Peruvian Amazon.

However, another predictive model was found in the literature, reinforcing our prediction model’s main arguments of simplicity, quickness, and non-invasiveness. In this case, it was developed for the early detection of metabolic syndrome (MS) and even shared some of the variables used for prediction, such as HBP [30].

### 4.3. Applicability in the Amazonian Context

Peru is a country that has, in macroeconomic terms, transitioned over the last few years from the group of low-income countries toward that of upper middle-income countries [31]. However, the Loreto region, where our study population is located, is far from the national average, and its characteristics are much more likely in a low-income country [32]. Indeed, the lower level of resources in this area highlights the value of the use of assessment tools such as the one developed in our study, since the combination of silent diseases and altered nutritional states, which can occur equally in high-income as in low-income settings such as the city of Iquitos, produce different functional forms. In other words, they have an unequal impact on quality of life and life expectancy, with more significant resources available for primary, secondary, and tertiary care [33]. Therefore, the tools and approaches used in the Western population should be adapted to people with the same characteristics as our sample, since their dietary patterns, lifestyles, and access to health care are radically different [34,35], making early diagnosis an aid in avoiding the need for care and treatment that are difficult to obtain.

### 4.4. Study Limitations

After analyzing the data, we considered that a larger sample should be available and contain data from both men and women to improve our model’s accuracy. The ethnic and socio-cultural characteristics of such an endemic area meant that, in general, the prevalence of variables detected was different from that expected. In addition, the percentage of DMT2 was very low, probably affecting the fact that despite the good AUC obtained, there were wide confidence intervals, and the R2 was not greater than 0.6. It was theorized that with a larger sample size, these model limitations could be overcome, in addition to determining the causality of the low prevalence of DMT2, especially important for the high intake of carbohydrates and sugars.

## 5. Conclusions

A model for predicting CR in a specific population of the Peruvian Amazon was developed based on independent anthropometric variables, nutritional status, and the presence of HBP or DMT2. The model’s advantages consist of its easy application by any health professional and, above all, without the need for blood tests or unavailable resources. The tool was used to measure CR, with results below expectations. In future studies, we will try to reach a larger sample size to refute the theory that the cutoff points proposed by the WHO to detect NCDs and obesity are not equally accurate for this population.

## Figures and Tables

**Table 1:** Sample description.

Variable	*n* or Mean (% or SD)
Age	42.9 (16.9)
Marital status	
Married	275 (75.5)
Single	89 (25.5)
Level of education	
None/Primary	279 (76.6)
Secondary/University	85 (23.4)
Employment status	
Employed	99 (27.3)
Unemployed	264 (72.7)
Nutritional status	
Normal weight	154 (42.2)
Overweight	136 (37.5)
Obesity	74 (20.3)
BMI (kg/m^2^)	26.5 (4.5)
WHtR	0.56 (0.06)
WC (cm)	88.9 (10.7)
WHR	0.96 (0.06)
ABSI	0.07 (0.005)
BAI	28.2 (4.3)
DFT (%)	25.5 (6.6)
HC (cm)	92.4 (7.5)

BMI: body mass index; WHtR: waist-to-height ratio; WC: waist circumference; WHR: waist-to-hip ratio; ABSI: a new body shape index; BAI: body adiposity index; DFP: Deuremberg fat percentage; HC: hip circumference.

**Table 2:** Bivariate analysis and logistic regression for Type 2 diabetes mellitus.

Variable	DMT2 Yes (*n* = 6)	DMT2 No (*n* = 357)	OR	IC95% OR	*p*
Age	46.2	42.9	1.01	(0.966–1.058)	0.633
Marital status					
Married	6 (2.2)	269 (97.8)	1		
Single	0 (0)	89 (100)	1.23		0.89
Level of education					
None/Primary	5(1.8)	274 (98.2)	1		
Secondary/University	1(1.2)	84 (98.8)	0.652	(0.075–5.662)	0.69
Employment status					
Employed	259 (98.1)	5 (1.9)	1		
Unemployed	98 (99)	1(1)	1.89	(0.218–16.4)	0.55
Anthropometric indexes (nutritional status)					
Normal weight	2 (1.3)	152 (98.7)	1		
Overweight	1 (0.7)	135 (99.3)	1.47	(0.267–8.161)	0.656
Obesity	3 (4.1)	71 (95.9)	1.11	(0.952–1.284)	0.190
BMI	28.9 (3.8)	26.4 (4.5)	3.37	(0.962–11.84)	0.057
WHtR	0.61(0.067)	0.55 (0.07)	1.06	(0.989–1.142)	0.100
WC	96.2 (9.91)	88.8 (10.7)	1.01	(0.996–1.005)	0.800
HC	97.17 (7.44)	92.34 (7.86)	1.078	(0.981–1.184)	0.11
WHR	0.98 (0.06)	0.95 (0.07)	1.11	(0.99–1.24	0.056
ABSI	0.08 (0.004)	0.07 (0.005)	2.34	(0.508–10.83)	0.275
BAI	31.7 (5.6)	28.1 (4.3)	1.15	(1.001–1.341)	<0.05
DFP	29.1 (7.2)	25.4 (6.6)	1.08	(0.96–1.22)	0.180
FINDRISC					
Low risk 0–14	4 (1.1)	347 (98.9)	1		<0.001
High risk 15–24	2 (15.4)	11 (84.6)	15.73	(2.606–95.45)	
Q2. NO family history of DM	2 (2.7)	71 (97.3)	1		0.291
Family history of DM	3 (1)	282 (99)	2.64	(0.43–16.14)	
Q4 > 30 min physical activity	2 (1)	194 (99)	1.35	(0.550–3.33)	0.550
<30 min physical activity	2 (1.9)	159 (98.1)	1		
Q5. Daily intake of fruits, vegetables, etc.	3 (1.1)	259 (98.9)	0.544	(0.09–3.3)	0.504
NO daily intake of fruits, vegetables	2 (2.1)	94 (97.9)	1		
Q6. No history of HBP drugs	4 (1.2)	318 (98.8)	1.5	(0.49–4.57)	0.470
History of HBP drugs	1 (2.8)	35 (97.2)	0.41		
Q3. WC no risk	2 (1.4)	138 (98.6)	1		
WC risk	4 (1.8)	220 (98.2)	1.255	(0.23–6.941)	0.795

Quantitative variables as mean and standard deviation. Qualitative variables as absolute frequency and percentage. BMI: body mass index; WHtR: waist-to-height ratio; WC: waist circumference; WHR: waist-to-hip ratio; ABSI: a new body shape index; BAI: body adiposity index; DFP: Deuremberg fat percentage; HC: hip circumference.

**Table 3:** Bivariate analysis and logistic regression for HBP.

Variable	HBP YES = 82 (22.52%)	HBP NO = 281 (77.2%)	*p*	OR	IC (18.09–26.95)	*p*
Age	52.8 (15.03)	40.07 (16.31)	0.001	1.04	(1.030–1.062)	0.001
Marital status						
Married	66 (21.1%)	208 (75.9%)	0.24	1		
Single	16 (18%)	73 (82%)		0.69	(0.376–1.268)	0.23
Level of education						
None/Primary	62 (22.3%)	216 (77.7%)	0.882	1		
Secondary/University	20 (23.5%)	65 (76.5%)		1.07	(0.603–1.905)	0.81
Employment status						
Employed	20 (20.3%)	79 (79.8%)	0.57	1		
Unemployed	62 (23.6%)	201 (76.4%)		1.21	(0.691–2.149)	0.495
Anthropometric indexes (nutritional status)						
Normal weight	22 (14.4%)	131 (85.6%)	0.001	1		
Overweight	32 (23.5%)	104 (75.6%)	0.001	2.38	(1.385–4.095)	0.005
Obesity	28 (51.85%)	26 (48.14%)	0.001	1.11	(1.048–1.167)	0.001
BMI	0.59 (0.061)	0.54 (0.067)	0.001	1.1	(1.04–1.16)	0.001
WHtR	94.79 (10.15)	87.22 (10.23)	0.001	1.1	(1.06–1.14)	0.001
WC	94.79 (1.12)	87.22 (0.61)	0.001	1.07	(1.04–1.09)	0.001
WHR	0.99 (0.059)	0.95 (0.067)	0.001	2.59	(1.7–3.9)	0.001
ABSI	0.08 (0.005)	0.07 (0.004)	0.001	3.09	(1.79–5.31)	0.001
BAI	29.61 (3.83)	27.75 (4.35)	0.001	1.1	(1.040–1.164)	0.001
DFP	29.69(5.8)	24.25(6.31)	0.001	1.14	(1.09–1.2)	0.001
FINDRISC						
Low risk 0–14	73(20.9%)	227 (79.1%)	0.001	1		
High risk 15–24	9 (69.2%)	4 (30.8%)		8.53	(2.55–28.5)	0.001
Q2. NO family history of DM	64 (22.5%)	221 (77.5%)	0.473	1		
Family history of DM	17 (23.6%)	55 (76.4%)		1.06	0.579–1.96)	0.83
Q4 > 30 min physical activity	162 (82.7%)	34 (17.3%)	0.005	1		
<30 min physical activity	47 (29.2%)	114 (70.8%)		1.96	(1.18–3.24)	0.008
Q5. Daily intake of fruits, vegetables, etc.	65 (24.9%)	196 (75.1%)	0.064	1		
NO daily intake of fruits, vegetables	16 (16.7%)	80 (83.3%)		1.65	(0.9–3.03)	0.102
Q6. No history of HBP drugs	57 (17.8%)	254 (82.2%)	0.001	1		
History HBP of drugs	24 (66.7%)	12 (33.3%)		3.04	(2.09–4.42)	0.001
Q3. WC no risk	36 (16%)	189 (84%)	0.001	1		
WC risk	43 (33.9%)	84 (66.1%)		2.68	(1.61–4.48)	0.001

Quantitative variables as mean and standard deviation. Qualitative variables as absolute frequency and percentage; BMI: body mass index; WHtR: waist-to-height ratio; WC: waist circumference; WHR: waist-to-hip ratio; ABSI: a new body shape index; BAI: body adiposity index; DFP: Deuremberg fat percentage; HC: hip circumference.

**Table 4:** Bivariate analysis and logistic regression for CR.

Variable	CR YES = 30 (8.24%)	CR NO = 333 (91.5%)	*p*	OR	IC (5.27–11.20)	*p*
Age	47.73 (12.91)	42.51(17.14)	0.105	1.04	(1.030–1.062)	0.001
Marital status						
Married	26 (9.5%)	248 (90.5%)	0.183	1		
Single	4 (4.5)	85 (95.5%)		0.69	(0.376–1.268)	0.23
Level of education						
None/Primary	23 (8.3%)	255 (91.7%)		1		
Secondary/University	7 (8.2%)	78 (91.8%)		1.07	(0.603–1.905)	0.81
Employment status						
Employed	3 (3%)	96 (97%)	0.03	1		
Unemployed	27 (10.3%)	236 (89.7%)		1.21	(0.691–2.149)	0.495
Anthropometric indexes (nutritional status)						
Normal weight	1 (0.7%)	152 (99.3%)	0.000	1		
Overweight	0	136 (100%)		2.38	(1.385–4.095)	0.002
Obesity	29 (39.2%)	45 (60.8)		1.11	(1.048–1.167)	0.001
BMI	33.05 (3.66)	25.89 (4.06)	0.001	1.1	(1.04–1.16)	0.001
WHtR	0.65 (0.04)	0.55 (0.065)	0.001	1.12	(1.048–1.167)	0.001
WC	103.88 (7.11)	87.58 (9.9)	0.001	1.07	(1.04–1.09)	0.001
WHR	1.01 (0.04)	0.95 (0.067)	0.001	1	(0.999–1.002)	0.645
ABSI	0.08 (0.003)	0.08 (0.005)	0.621	3.09	(1.798–5.319)	0.001
BAI	32.81 (3.47)	27.75 (4.15)	0.001	1.1	(1.040–1.164)	0.001
DFP	34.44(5.15)	24.67(6.1)	0.001	1.32	(1.21–1.44)	0.001
FINDRISC						
Low risk 0–14				1		
High risk 15–24	22 (6.3%)	328 (93.7%)	0.001	8.53	(2.55–28.5)	0.001
Q2. NO family history of DM	8 (61.5%)	5 (38.5%)	0.001	1		
Family history of DM	20 (7%)	365 (93%)	0.104	1067	0.579–1.96)	0.83
Q4 > 30 min physical activity	9 (12.5%)	63 (87.5%)	0.104	1		
<30 min physical activity	13 (6.6%)	183 (93.4%)	0.173	1.96	(1.18–3.24)	0.008
Q5. Daily intake of fruits, vegetables, etc.	16 (9.9%)	145 (90.1%)	0.173	1		
NO daily intake of fruits, vegetables	23 (8.8%)	238 (91.2%)	0.292	1.65	(0.9–3.03)	0.102
Q6. No history of HBP drugs	6 (6.3%)	90 (93.8%)	0.292	1		
History of HBP drugs	18 (5.6%)	303 (94.4%)	0.001	3.04	(2.09–4.42)	0.001
Q3. WC no risk	11 (30.6%)	25 (69.4%)	0.001	1		
WC risk	2 (0.6%)	331 (99.4%)	0.001	2.68	(1.61–4.48)	0.001

Quantitative variables as mean and standard deviation. Qualitative variables as absolute frequency and percentage. BMI: body mass index; WHtR: waist-to-height ratio; WC: waist circumference; WHR: waist-to-hip ratio; ABSI: A new body shape index; BAI: body adiposity index; DFP: Deuremberg fat percentage; HC: hip circumference.

**Table 5:** Comparison of logistic regressions adjusted for CR.

Cardiovascular Risk	ß	OR	IC 95%	*p*	r^2^	Hosmer and Lemshow	AUC
Model 1					0.62	0.292	0.92
SBP (qualitative)	4.04	56.8	(15.16–214.21)	0.001			
HC	0.234	1.26	(1.15–1.38)	0.001			
Model 2					0.54	0.95	0.94
SBP (quantitative)	0.076	1.07	(1–05-1.1)	0.001			
WC	0.233	1.26	(1.16–1.36)	0.001			
Model 3					0.53	0.76	0.93
SBP	0.059	1.06	(1.03–1.08)	0.001			
WC	0.171	1.18	(1.11–1.26)	0.001			
Model 4					0.57	0.92	0.94
SBP (quantitative)	0.74	1.08	(1.05–1.1)	0.001			
HC	0.21	1.24	(1.14–1.34)	0.001			
FINDRISC (LOW/HIGH RISK)	2.06	7.86	(1.42–43.54)	0.018			

**Table 6:** Comparison of diagnostic accuracy between the two selected models (1 and 4).

	Model 1		Model 4	
Diagnostic Test	Value	CI95%	Value	CI95%
Sensitivity (%)	86.96	95.12–99.1	73.68	81.25–96.12
Specificity (%)	97.06	94.37–98.47	95.35	92.98–97.72
Validity index (%)	96.42	94.37–98.47	94.21	91.68–96.75
Predictive value + (%)	66.67	48.13–85.2	46.67	27.16–66.19
Predictive value − (%)	99.10	97.93–100	98.50	97.04–99.95
Prevalence (%)	6.34	3.69–8.98	5.23	2.81–7.66
Youden index	0.84	0.70–0.98	0.69	0.49–0.89

## Data Availability

The data presented in this study are available on request from the corresponding author. The data are not publicly available due to company rules.
